# Symptomatic pelvic floor disorders and its associated factors in South-Central Ethiopia

**DOI:** 10.1371/journal.pone.0254050

**Published:** 2021-07-01

**Authors:** Eskedar Demissie Beketie, Wubishet Tesfaye Tafese, Zebene Mekonnen Assefa, Fantahun Walle Berriea, Genet Asfaw Tilahun, Bisrat Zeleke Shiferaw, Natnael Eshetu Teke

**Affiliations:** Department of Nursing, College of Medicine and Health Sciences, Wolkite University, Wolkite, Ethiopia; University Medical Center Utrecht, NETHERLANDS

## Abstract

**Introduction:**

Pelvic floor disorders (PFD) are gynecologic health problems containing a wide variety of clinical problems; the most prevalent problems are pelvic organ prolapse, fecal incontinence, and urinary incontinence. It is a significant women’s health problem for both developed and developing countries. One in five women in Ethiopia experiences at least one major type of pelvic floor disorders. Despite the severity of the problem, due attention was not given, and no study has been conducted on pelvic floor disorders in the Gurage Zone.

**Objective:**

To determine the prevalence and associated factors of symptomatic pelvic floor disorders among women living in Gurage Zone, SNNPR, Ethiopia, 2020.

**Methodology:**

Community-based cross-sectional study was conducted from February to March 2020 among 542 women residing in the Gurage Zone. A multi-stage sampling method was used to select the participants. Interviewer administered, pretested questionnaires containing questions related to pelvic organ prolapse, urinary, and fecal incontinence was used. The urinary incontinence severity index questionnaire was used to assess the severity of urinary incontinence. Epi-Info x7 was used to record data, and SPSS was used to analyze the data. Binary logistic regression with 95% CI was used to explore the relationship between PFD and other independent variables. After multivariable logistic regression analysis variables with P-value less than 0.05 was used to determine significant association.

**Result:**

A total of 542 participants were included in this study. Overall, 41.1% of the participants reported one or more symptoms of pelvic floor disorders. Urinary incontinence had the highest prevalence (32.8%), followed by pelvic organ prolapse (25.5%) and fecal incontinence (4.2%). History of weight lifting >10 Kg (AOR = 3.38; 95% CI: 1.99, 5.72), ≥5 vaginal delivery (AOR = 11.18; 95% CI: 1.53, 81.58), and being in menopause (AOR = 3.37; 95% CI: 1.40, 8.07) were identified as possible contributing factors in the development of a pelvic floor disorders.

**Conclusion:**

The prevalence of symptomatic PFD was higher compared to other similar studies in Ethiopia. Heavy weight lifting, repetitive vaginal deliveries and menopause were factors significantly associated with PFD. Expansion of technologies and building basic infrastructures, health education on kegel exercise and promotion of family planning should be considered as a prevention strategy.

## Introduction

Pelvic floor disorders (PFD) are a gynecologic health problems containing a wide variety of clinical diagnoses. The most prevalent problems are pelvic organ prolapse (POP), fecal incontinence (FI), and urinary incontinence (UI) and their symptoms can range from slight embarrassment to intolerable social and psychological problems [1–3]. The International Urogynecological Association (IUGA) and International Continence Society (ICS) jointly defined UI as the complaint of any involuntary loss of urine and FI as a complaint of involuntary loss of solid or liquid feces. According to the IUGA and ICS joint definition, POP is a symptomatic descent of one or more of the posterior vaginal wall, anterior vaginal wall, and the apex of the vagina or vault after hysterectomy [1].

Pelvic floor disorders are common debilitating health problems among women throughout the world. One in every four women suffer from pelvic floor disorders in developed countries [4]. Even though there are not enough studies to conclude about the prevalence of PFD in developing countries, it could be higher than the developed ones because of poor nutrition, early marriage, high parity, vaginal delivery and heavy weight lifting [5, 6]. Due to lack of knowledge, socio-cultural view, and fear of discrimination, many women with PFD do not disclose their health problem; however, they are a significant health problems among women living in low and middle-income countries, in which the prevalence of POP ranges from 3.4%–56.4%, UI ranges from 5.2%–70.8%, and FI ranges from 5.3% to 41.0% [3, 5]. Similarly, in Ethiopia, one in eight to one in every five women experiences at least one type of pelvic floor disorders and POP is among the most common indications for gynecological surgery [7–9]. According to a one-year review of POP at St Paul’s Hospital Millenium Medical College in Addis Ababa, 57% of patients with POP operated at the hospital were Gurage in ethnicity [10].

Pelvic floor disorders can result in multiple adverse outcomes in the daily life of a woman. Most women with PFD feel extreme shame, humiliation and anxiety because of their health status. Women with advanced POP and UI have decreased body image, have a lower quality of life, and some may isolate themselves from society. It also hinders them from performing their daily activity like sitting down for the toilet, walking long distances, or lifting heavy materials. About 43% of women with PFD will experience sexual dysfunction. Many women with advanced POP experience conflict in their marriage and some of them end up in divorce due to sexual dysfunction. About 67.7% of women with advanced POP are depressed. They are also victims of social discrimination when disclosing their health problems [2, 3, 11–13].

Despite the severity of the problem, PFD are less well studied women’s health problem in Ethiopia. Only a few studies have been conducted on the problem and most of them mainly focus on a single PFD, commonly POP. Urinary incontinence and FI were the least studied topics among PFD. There are no studies in the Gurage Zone specifically. This study will assess the prevalence and factors associated with symptomatic PFD among women reside in Gurage Zone.

## Materials and methods

### Study area and period

A community-based cross-sectional study was used to assess the prevalence and associated factors of symptomatic pelvic floor disorders among women living in the Gurage Zone from February 01, 2020, to March 30. Gurage Zone is located in the Southern nations, nationalities and peoples regional sate (SNNPR) of Ethiopia. It is bordered on the southeast by Hadiya and Yem special woreda, on the west, north and east by the Oromia Region, and on the southeast by Silt’e. It is administratively organized into 16 Woredas and 5 administrative towns. Each Woreda is further subdivided into different kebeles, which are the smallest formal administrative units. Kocho is a traditional food, which is processed from enset plant and it is a very important local food source for Gurage people.

### Population

#### Source population

The source population was all women living in Gurage zone.

#### Study population

All women who resided in the selected kebeles of Gurage zone was considered as a study population.

#### Eligibility criteria

Women who lived for at least 6 months in Gurage zone were eligible for the study. Girls less than 18 years of age who could not give consent, women who are unable to listen or speak and those with serious medical condition were excluded from the study.

### Sample size determination

The sample size was determined using a single population proportion formula, taking an assumption of 95% confidence level, 0.05 margin of error, and a 20.5% proportion of PFD [14]. Adding a 10% nonresponse rate, the sample size was calculated to be 276. Since a multi-stage sampling technique was used, the sample size was multiplied by 2, giving the final sample size of 553.

### Sampling procedure

**Fig 1:**
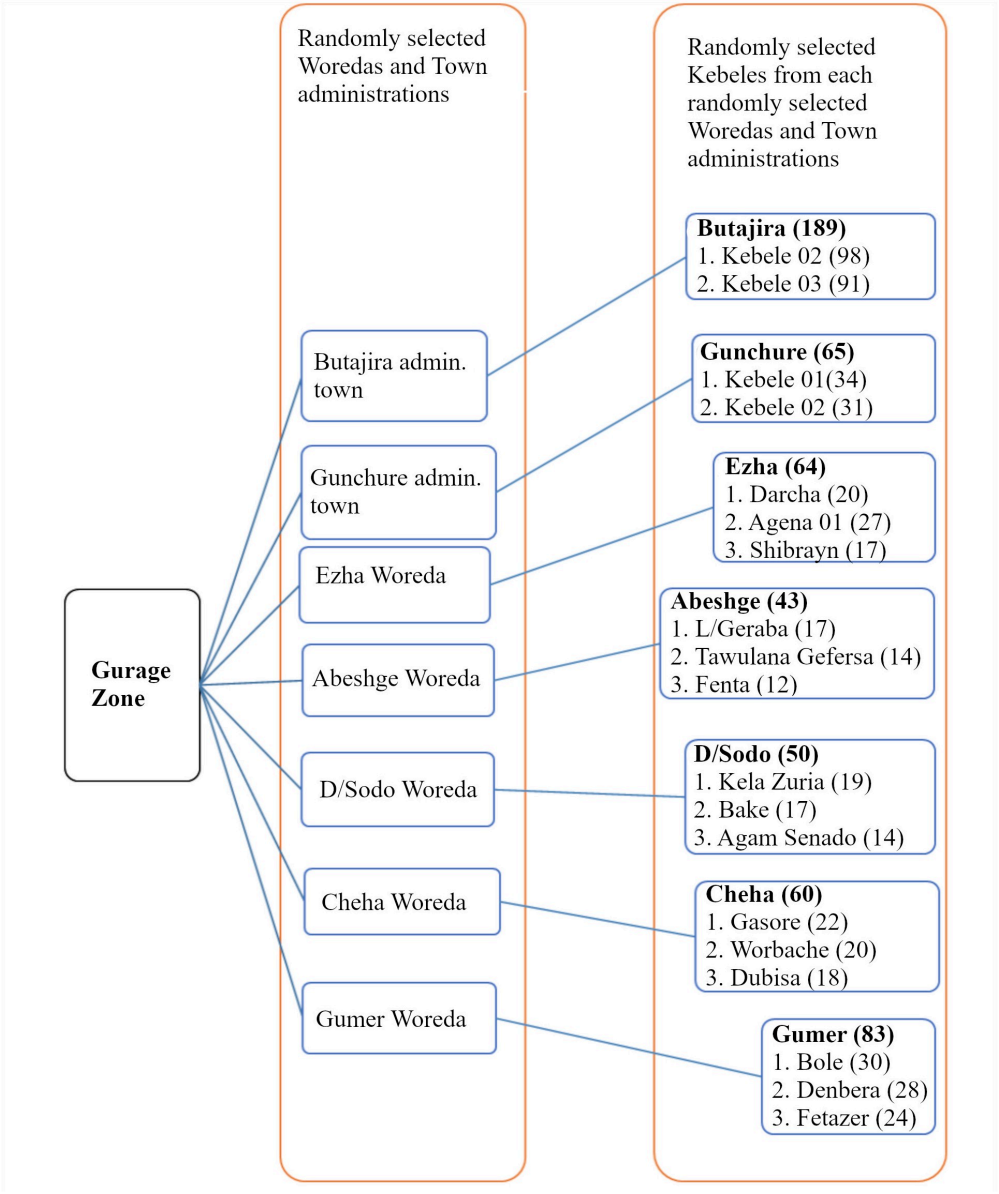
Schematic presentation of sampling technique to assess the prevalence and associated factors of symptomatic pelvic floor disorders among women living in Gurage Zone, SNNPR, Ethiopia, 2020.

The Gurage Zone has 16 woredas and five administrative towns. We took five woredas and two administrative towns randomly by a lottery method. Considering homogeneity within the administrative towns and woredas, we took three kebeles from each randomly selected woredas, and we took two kebeles from each administrative towns. Then the sample size was allocated for each kebeles proportionally based on the number of households they have (Fig 1). Finally, systematic random sampling technique was used to select the households and eligible woman was recruited from each. When there are more than one woman in a household who can fulfill our eligibility criteria, we used a lottery method to select one of them.

### Study variables

#### Dependent variables

Pelvic floor disorders.

#### Independent variables

The independent variables are: Socio-demographic variables (i.e. Age, marital status, education, occupation, residence, Income), general health condition (i.e. BMI, recurrent UTI, Diabetes mellitus, depression, lung disease, neurologic disease, use of diuretics, chat chewing, caffeine, weight lifting >10Kg), and Obstetric and gynecologic factors (i.e. gravidity, parity, miscarriage, current pregnancy, place of delivery, age at first delivery, mode of first delivery, number of vaginal deliveries, vacuum/forceps delivery, episiotomy, perineal tear, CS, VBAC, longest labor, menopause, hysterectomy, contraceptive, family history of PFD).

### Data collection tool and measurement

Data were collected using an interviewer-administered, pretested questionnaire containing questions related to urinary and fecal incontinence according to the definition given by IUGA and ICS [1]. A woman was considered to have urinary incontinence if any of the following conditions were confirmed. Stress urinary incontinence: when there was involuntary leakage of urine on activities like effort or exertion, or on sneezing or coughing; urge UI: when there was involuntary leakage which go along with or immediately preceded by urgency; and mixed UI: when there was involuntary leakage of urine associated with both urgency and stress UI. Similarly, fecal incontinence was considered present in women with any involuntary loss of solid or liquid fecal matter at least monthly over the last1year.

Pelvic organ prolapse was assessed by two questions used in a study done in the USA and the pilot study in Ethiopia: "Do you have a feeling of bulging/pressure, or something seems to be coming down through the vagina?", and "Do you have visible mass protruding via the vagina?" [7, 15]. If a woman confirmed the presence of any of the above two questions, she was considered to have symptomatic POP. For the purpose of this study, women who had at least one of the UI, FI, and POP were grouped as having symptomatic PFD.

The urinary incontinence severity index was also used to assess the severity of urinary incontinence. It contains two questions that ask the amount and frequency of urinary leakage. The frequency has four values and the amount has two. By multiplying the score from the two questions, score 1–2 were considered to have light UI, 3–4 moderate UI, and 6–8 severe UI [16]. Recurrent urinary tract infection (UTI) was considered if a participant reported to be diagnosed by a clinician three or more times in the last 12 months. Questions that assess other variables like socio-demographic factors, obstetric and gynecologic factors, and medical health problems were mainly adopted from the Epidemiology of prolapse and incontinence questionnaire (EPIQ) [17].

### Data collection procedure

Ten Bachelor of Science degree holder midwives/ nurses who were fluent in English and the local languages (Amharic and Guragigna) were hired as data collectors. Two days of training on the interviewing technique, the study’s objective, and different questionnaire sections were given. Two supervisors were also hired to monitor the process of data collection and quality of data collected. Each questionnaires were checked by the supervisors at the end of each day’s data collection. A pretest was done on 5% (28 women) of the total sample size before the actual data collection. Some minor edition was undertaken based on a pretest result.

The data was collected on home to home basis. The data collectors interview the participants and record their responses on a given questionnaire.

### Data analysis

Data were recorded in Epi Info-7 and then transferred to SPSS version 23 for further analyses. Summary statistics like frequencies, percentage, median and inter-quartile range (IQR) was calculated using descriptive statistics function of SPSS. Binary logistic regression with 95% CI was used to explore the relationship between PFD and other independent variables. Those variables with a p-value of less than 0.15 were inserted into a multivariable logistic regression analysis to see their independent effect. Variables with a p-value of less than 0.05 were considered significant statistical associations with the dependent variable. Model fitness was checked by Hosmer-Lemeshow goodness of fit test.

### Ethical considerations

The study obtained ethical approval from Wolkite University’s Institutional Review Board (IRB), and permission was obtained from the respective kebele administrators. Moreover, written consent was obtained from each study participant before the commencement of data collection. Participant information sheet and consent form has been attached with the questionnaire. Participants who cannot read and write gave consent after the interviewer read the information to them. The interviewer only proceeds after a consent to participate has been approved. To maintain confidentiality, participant’s identification was not recorded. Likewise, results were also analyzed and presented in aggregate.

## Results

### Socio-demographic characteristics

Five hundred fifty-three women were approached, and 542 participated in this study giving a 98% response rate. The age of the participants ranged from 19–70 and the median age was 36 (IQR = 20). Most (74.5%) of them were married and more than half (53.5%) were housewives (Table 1). More than half (55.2%) of the participants reported doing heavy physical work in the present or past. They mentioned fetching water from a distance using more than 20L containers, collecting and slicing firewood, processing kocho, carrying heavy objects to and from the market, and other farming activities as heavy physical work in their day-to-day life.

**Table 1:** Socio-demographic characteristics of women living in Gurage Zone, SNNPR, Ethiopia, 2020.

Socio-demographic Variable (N = 542)		Frequency	Percent (%)
Age*	15–24	68	12.5
	25–34	162	29.9
	35–44	126	23.2
	45–54	114	21.0
	55+	72	13.3
Ethnicity	Gurage	406	74.9
	Amhara	43	7.9
	Kabena	35	6.5
	Oromo	27	5.0
	Others (Wolita, Hadiya, Yem)	31	5.7
Marital status	Never Married	50	9.2
	Married and living together	404	74.5
	Divorced/Separated	36	6.6
	Widowed	52	9.6
Educational status	Unable to read and write	193	35.6
	Able to read and write	86	15.9
	Primary (1–8)	102	18.8
	Secondary (9–12)	82	15.1
	College or Above	79	14.6
Occupation	Housewife	290	53.5
	Farmer	34	6.3
	Employee	82	15.1
	Merchant	102	18.8
	Other (Daily laborer, students)	34	6.3
Residence	Urban	223	41.1
	Rural	319	58.9
Heavy physical work	Yes	299	55.2
	No	243	44.8
Income** (N = 489)	< 2000	183	37.4
	2000–2999	134	27.4
	3000–4999	111	22.7
	5000+	61	12.5

*Age in years.

** Income in Ethiopian Birr (ETB).

### General health condition

Three-quarter (75.2%) of the participants’ BMI was in the normal range (18.5–25 Kg/m^2^). Fifty-four (10.0%) were known diabetic patients, and 264 (48.7%) responded yes for heavyweight lifting (>10Kg) regularly (Table 2).

**Table 2:** General health condition of women living in Gurage Zone, SNNPR, Ethiopia, 2020.

General health-related Variables (N = 542)		Frequency	Percent (%)
**BMI (N = 528)**	< 18.5	74	14.0
	18.5–25	397	75.2
	>25	57	10.8
**Recurrent UTI**	Yes	91	16.8
	No	451	83.2
**Diabetes Mellitus**	Yes	54	10.0
	No	488	90.0
**Depression**	Yes	10	1.8
	No	532	98.2
**Lung disease**	Yes	86	15.9
	No	456	84.1
**Neurologic disease**	Yes	6	1.1
	No	536	98.9
**Use of diuretics**	Yes	28	5.2
	No	514	94.8
**Chat Chewing**	Yes	42	7.7
	No	500	92.3
**Caffeine**	Yes	479	88.4
	No	63	11.6
**Weight lifting (>10Kg)**	No	220	40.6
	Yes	264	48.7
	I do not know	58	10.7
**Years of weight**	<10	87	16.9
**Lifting (N = 264)**	11–20	110	22.7
	21+	67	13.8

### Obstetric and gynecologic history

Seventy four (13.7%) of the women were nulliparous, and 77 (14.2%) were pregnant at the time of data collection. One hundred three (22.8%) delivered a baby before their twentieth birthday. Of the 74 mothers who had a history of cesarean section (CS), 10 (13.5%) tried vaginal birth after cesarean section (VBAC) (Table 3).

**Table 3:** Obstetric and gynecologic information of women living in Gurage Zone, SNNPR, Ethiopia, 2020.

Variables	N		Frequency	Percent (%)
**Gravidity**	542	0	68	12.5
		1–4	244	45.0
		5+	230	42.4
**Parity**	542	Nulliparous	74	13.7
		1–4	292	53.7
		5+	176	32.7
**Miscarriage**	542	Yes	165	30.4
**Current Pregnancy**	542	Yes	77	14.2
**Trimester of pregnancy**	46	First trimester	5	10.9
		Second trimester	27	58.7
		Third trimester	14	30.4
**Place of delivery**	468	All at home	89	19.0
		Both	170	36.3
		All at health institution	209	44.7
**Age at first delivery**	452	<18	103	22.8
		≥18	349	77.2
**Mode of first delivery**	468	Vaginal	438	93.6
		Cesarean section	30	6.4
**delivered vaginally**	542	Yes	449	82.8
**Number of vaginal deliveries**	542	0	93	
		1–4	279	
		5 or more	170	
**Forceps or vacuum delivery**	449	Yes	36	8.0
		No	404	90.0
		I do not know/remember	9	2.0
**Episiotomy**	449	Yes	195	43.4
		No	248	55.2
		I do not know/remember	6	1.3
**Tear**	449	Yes	56	12.5
		No	358	79.7
		I do not know	35	7.8
**CS delivery**	458	Yes	74	15.8
**Trial of VBAC**	74	Yes	10	13.5
**Longest labor**	335	< = 12	85	25.4
		13–24	222	66.3
		25+	28	8.4
**Menopause**	542	Yes	112	20.7
		No	424	78.2
		I do not know	6	1.1
**Hysterectomy**	542	Yes	23	4.2
**Contraceptive**	542	Yes	164	30.3
**Family history of PFD**	542	Yes	118	21.8
		No	401	74.0
		I do not know	23	4.2

VBAC- Vaginal birth after cesarean.

CS- cesarean section.

### Prevalence of pelvic floor disorders

Overall, 41.1% (95% CI: 36.9, 45.3) of the participants reported symptoms of one or more pelvic floor disorders. Urinary incontinence being the most highly reported type of PFD, 178 (32.8%) of all the participants reported the symptoms. Pelvic organ prolapse, and fecal incontinence accounts 138 (25.5%) and 23 (4.2%), respectively.

### Associated factors

All variables were computed separately on bivariabe logistic regression, and those variables with p-value less than 0.15 were considered for multivariable logistic regression analysis. The model was well fit according to a Hosmer-Lemeshow goodness of fit test (sig. = 0.236). Gravidity, parity, and number of vaginal deliveries have been inserted into linear logistic regression for collinearity diagnostic. Based on this test, the Variance inflation factor (VIF) was 6.205, 11.113 and 7.802 for gravidity, parity, and number of vaginal deliveries respectively. Hence the number of vaginal deliveries was included in the regression model for further analysis with the other ten variables. Among the eleven variables entered into a multivariable logistic regression, three showed a significant association. According to this, history of weight lifting greater than 10 Kg (AOR = 3.38; 95% CI: 1.99, 5.72), ≥5 vaginal delivery (AOR = 11.18; 95% CI: 1.53, 81.58), and being in menopause (AOR = 3.37; 95% CI: 1.40, 8.07) were the factors associated with pelvic floor disorders (Table 4).

**Table 4:** Logistic regression analysis showing the association between symptomatic pelvic floor disorders and other factors among women living in Gurage Zone, SNNPR, Ethiopia, 2020.

Variables	PFD		COR (95% CI)	AOR (95% CI)
	Yes 223(%)	No 319(%)		
**Age**				
15–24	4(1.8)	64(20.1)	1	1
25–34	60(26.9)	102(32.0)	9.41(3.26, 27.15)	1.86(0.53, 6.58)
35–44	44(19.7)	82(25.7)	8.59(2.93, 25.14)	1.01(0.28, 3.68)
45–54	70(31.4)	44(13.8)	25.46(8.66, 74.82)	1.14(0.30, 4.38)
55+	45(20.2)	27(8.5)	26.67(8.73, 81.50)	0.83(0.16, 4.27)
**Marital status**				
Never Married	3(1.3)	47(14.7)	1	1
Married and living together	174(78.0)	230(72.1)	11.85(3.63, 38.71)	1.08(0.20, 5.80)
Divorced/separated	16(7.2)	20(6.3)	12.53(3.28, 47.84)	0.96(0.15, 6.12)
Widowed	30(13.5)	22(6.9)	21.36(5.88, 77.63)	0.92(0.15, 5.77)
**Educational status**				
Unable to read and write	102(45.7)	91(28.5)	1	1
Able to read and write	46(20.6)	40(12.5)	1.03(0.62, 1.71)	1.59(0.84, 3.00)
Primary	49(22.0)	53(16.6)	0.83(0.51, 1.33)	1.23(0.64, 2.39)
Secondary	16(7.2)	66(20.7)	0.22(0.12, 0.40)	0.66(0.28, 1.59)
College or above	10(4.5)	69(21.6)	0.13(0.06, 0.27)	0.59(0.21, 1.64)
**Occupation**				
Housewife	137(61.4)	153(48.0)	1	1
Farmer	15(6.7)	19(6.0)	0.88(0.43,1.80)	0.98(0.38,2.48)
Employee	13(5.8)	69(21.6)	0.21(0.11,0.40)	0.69(0.29,1.67)
Merchant	53(23.8)	49(15.4)	1.21(0.77,1.90)	1.65(0.90,3.04)
Others(Daily laborers, students)	5(2.2)	29(9.1)	0.19(0.07,0.51)	0.43(0.11,1.75)
**Lung Disease**				
No	177(79.4)	279(87.5)	1	1
Yes	46(20.6)	40(12.5)	1.81(1.14,2.88)	1.64(0.91,2.96)
**History of weight lifting (>10Kg)**				
No	58(26.0)	162(50.8)	1	1
Yes	148(66.4)	116(36.4)	3.56(2.42,5.24)	3.38(1.99,5.72) *
I Do not Know	17(7.6)	41(12.9)	1.16(0.61,2.20)	1.18(0.55,2.55)
**Ever had miscarriage**				
No	130(58.3)	246(77.1)	1	1
Yes	93(41.7)	73(22.9)	2.41(1.66,3.50)	1.39(0.85,2.26)
**Age at first delivery**				
<18	64(30.2)	39(16.3)	1	1
≥18	148(69.8)	201(83.8)	0.41(0.28,0.61)	1.14(0.64,2.05)
**Mode of first delivery**				
Vaginal	208(95.4)	230(92.0)	1	1
CS	10(4.6)	20(8.0)	0.55(0.25,1.21)	3.93(0.84,18.38)
**No of vaginal delivery**				
0	9(4.0)	84(26.3)	1	1
<5	108(48.4)	171(53.6)	5.89(2.85,12.21)	6.12(0.90,41.69)
≥5	106(47.5)	64(20.1)	15.46(7.27,32.86)	11.18(1.53,81.58) *
**Menopause**				
No	146(66.4)	278(88.0)	1	1
Yes	74(33.6)	38(12.0)	3.71(2.39,5.75)	3.37(1.40,8.07) *

* Significantly associated factors.

1: Reference.

COR: crude odds ratio.

AOR: adjusted odds ratio.

## Discussion

According to this study, the prevalence of symptomatic PFD was 41.1%. This finding was lower as compared to studies conducted in Turkey (67.5%) and Australia (47.2%) [18, 19]. The inconsistency can be explained by the difference in the study population and sample size. The Australian study included only women aged 65–79 years. On the other hand, the prevalence of PFD in the current study was higher as compared to studies in the USA (25%), Bangladesh (35.5%), India (21%), Dabat (11.9%), and Kersa (20.5%) [4, 7, 10, 20, 21]. The variation in the study population can partly explain this difference. A higher proportion of grand multiparity and vaginal delivery in the current study compared to studies from India and the USA might explain this discrepancy. The study in the USA excluded slight UI based on the severity index. The higher prevalence of PFD in the current study compared to studies in Dabat and Kersa can be justified by the high proportion of women who did heavy physical work, including lifting heavy objects and processing kocho/enset in the current study. Overall, UI and POP accounts for 32.8% and 25.5%, respectively. The results were slightly higher than the mean prevalence found in a review by Walker and Gunasekera on the prevalence and risk factors of pelvic organ prolapse and incontinence in developing countries, which states 28.7% and 19.7% for UI and POP correspondingly. According to the review, the prevalence of UI ranges from 5.2–70.8%, and POP ranges from 3.4–56.4%, in which the prevalence of the current study fall. The mean prevalence of FI reported in the review was higher compared to this study [5]. On the other hand it is in line with a systemic review and meta-analysis in low and middle-income countries [22]. For local authorities and other concerned bodies, we recommend planning for regular campaigns to screen and link the women to health institution for further diagnosis and treatment of the problem. Training health extension workers on common risk factors and on how to screen and link suspected cases to nearby hospitals could support the effort in reducing the risks and treating cases of PFD.

Studies using physical examination to assess POP showed a higher prevalence of POP than the current study. The POP questions used in the current study had a high correlation with stage two or more severe stage of POP based on Pelvic Organ Prolapse Quantification exam (POP-Q) and the questions had high specificity than sensitivity [7, 15]. In addition to these aspects, such kinds of problems are considered embarrassing. So, many women do not want to share their health condition. These reasons might explain the low proportion of symptomatic POP compared to those studies that used the objective method of POP assessment [7, 23]. Using only a questionnaire to assess POP can be mentioned as a limitation of this study, because the tool might miss mild cases. A direction for future research would be the use of an objective assessment method (physical examination) in addition to an interview questionnaire in the study population of interest.

According to the severity index, we found 7.4% had moderate to severe urine incontinence, while 25.5% had slight incontinence. The prevalence of urge and stress incontinences was 5.1% and 12.9%, respectively, and 14% of women had mixed type. In most part, this result was in-line with a study done in Norway, which reported 8.7% for the prevalence of moderate or severe incontinence and 12.2% for stress incontinence. The prevalence of urge incontinence and mixed type was 1.8%, and 5.9% respectively, which was relatively lower than the current study. This difference might be because the Norwegian study excluded women of age greater than 65, those who had more than four vaginal deliveries and those who had a history of both vaginal and cesarean delivery.

This study showed that women who lifts weight more than 10 kg on a regular basis had an increased risk of developing PFD symptoms. This was consistent with studies conducted in Sweden, Dabat, Bahirdar, Mizan, and a systemic review conducted in low and middle-income countries [5–7, 24, 25]. Heavy physical work and heavy weight lifting increase intra-abdominal pressure, which is believed to play a role in the pathogenesis of PFD (specifically POP) [26, 27]. In the current study, more than half of the women live in rural areas and most of them are engaged in heavy physical work like fetching water using more than 20L containers from a distance, collecting and slicing firewood, farming, carrying goods to and from the market, and other energy demanding and straining activities. As kocho is traditional and favorite food in the study area, processing kocho/enset is also one of the heavy tasks women have to perform. Expansion of technologies, like machineries that can help with processing kocho and building basic infrastructures to modify the lifestyle of women should also be considered as a strategy to reduce PFD.

According to the current study, mothers who had five or more vaginal deliveries had 11.2 times higher odds of having PFD compared to those who had no vaginal delivery. This is comparable with a study done in Kersa, which indicated that five or more vaginal deliveries was associated with PFD compared to four or less vaginal delivery. In the same way, studies conducted in the USA, Sweden, and Bahirdar reported multiparous women with ≥ 4 parity had a greater risk of developing PFD than nulliparous women [4, 24, 25]. Similarly, two cohort studies [28, 29] indicated that vaginal delivery was significantly associated with an increased risk of incontinence and prolapse compared with women only having cesarean deliveries. Another prospective study among 5000 black women in sub-Saharan Africa also reported women with a history of vaginal delivery were approximately two-fold more likely to report urine leakage [30]. The association of repetitive vaginal delivery with PFD may result from direct injury to the pelvic muscles and connective tissue. With repeat vaginal delivery, direct injury to the pelvic muscles, connective tissue, and nerve damage due to trauma and excessive stretching could result in pelvic floor dysfunction [26]. As high parity is a common trend in developing countries, we recommend to reduce number of pregnancy and hence reduce the risk of PFD due to excessive stretch and trauma during child birth. To achieve this, the national and international effort towards family planning should be strengthened.

In this study, postmenopausal women were more likely to report symptoms of PFD. The physiologic process of aging, degenerative process, and hypo-estrogenism creates urogenital atrophy and weakens pelvic organ supporting structures. As a consequence, the risk of PFD will be increased. Reproductive hormones may be crucial in normal urinary function and maintenance of connective tissue that supports the pelvic organs [26, 31]. This might explain the association between PFD and menopause. We recommend to consider estrogen replacement therapy for postmenopausal women when necessary. Additionally, health institutions and other concerned bodies should promote and provide health education on Kegel exercise for women. Kegel exercise may reduce the risk of PFD by strengthening the pelvic floor muscles.

Many studies report BMI was an associated factor, but BMI was not significantly associated with PFD in the current study. Compared to studies in Bahirdar and the USA, most of the current study participants were of normal BMI. A smaller proportion of underweight women in our study compared to the study from Bahirdar and a smaller proportion of overweight/obese women compared to the study from USA may explain the difference [25, 32]. But other studies are in line with the current study [7, 21].

## Conclusion

The prevalence of symptomatic PFD was higher as compared to other studies in Ethiopia. Heavy weight lifting, repetitive vaginal deliveries and being in menopause were factors significantly associated with PFD.

Expansion of technologies, like machineries that can help with processing kocho and building basic infrastructures to modify the lifestyle of women should also be considered as a prevention strategy. In addition health education on kegel exercise and promotion of family planning could also be beneficial.
